# Circulating Exosomal miR-1290 for Diagnosis of Epithelial Ovarian Cancer

**DOI:** 10.3390/cimb44010021

**Published:** 2022-01-09

**Authors:** Hyeji Jeon, Su Min Seo, Tae Wan Kim, Jaesung Ryu, Hyejeong Kong, Si Hyeong Jang, Yong Soo Jang, Kwang Seock Kim, Jae Hoon Kim, Seongho Ryu, Seob Jeon

**Affiliations:** 1 Department of Obstetrics and Gynecology, College of Medicine, Soonchunhyang University, Cheonan Hospital, Cheonan 31151, Korea; hjjeon@schmc.ac.kr (H.J.); marqu2moon@naver.com (Y.S.J.); 2 Soonchunhyang Innovative Convergence Research Center, Soonchunhyang University, Cheonan Hospital, Cheonan 31151, Korea; ktwdreem@naver.com (T.W.K.); rjs652@naver.com (J.R.); angelkonghj@gmail.com (H.K.); kimks5005gt@gmail.com (K.S.K.); 3 Soonchunhyang Institute of Med-Bio Sciences (SIMS), Soonchunhyang University, Cheonan 31151, Korea; 4 Department of Medical Life Science, Soonchunhyang University, Asan 31538, Korea; 5 Department of Pathology, College of Medicine, Soonchunhyang University, Cheonan Hospital, Cheonan 31151, Korea; 82632@schmc.ac.kr; 6 Department of Obstetrics and Gynecology, Gangnam Severance Hospital, Yonsei University College of Medicine, Seoul 05029, Korea; jaehoonkim@yuhs.ac.kr

**Keywords:** ovarian cancer, exosomal microRNA, biomarker, early diagnosis

## Abstract

The aim of the study was to develop a new diagnostic biomarker for identifying serum exosomal miRNAs specific to epithelial ovarian cancer (EOC) and to find out target gene of the miRNA for exploring the molecular mechanisms in EOC. A total of 84 cases of ovarian masses and sera were enrolled, comprising EOC (*n* = 71), benign ovarian neoplasms (*n* = 13). We detected expression of candidate miRNAs in the serum and tissue of both benign ovarian neoplasm group and EOC group using real-time polymerase chain reaction. Immunohistochemistry were constructed using formalin fixed paraffin embedded (FFPE) tissue to detect expression level of suppressor of cytokine signaling 4 (SOCS4). In the EOC group, miRNA-1290 was significantly overexpressed in serum exosomes and tissues as compared to benign ovarian neoplasm group (fold change ≥ 2, *p* < 0.05). We observed area under the receiver operating characteristic curve (AUC) for miR-1290, using a cut-off of 0.73, the exosomal miR-1290 from serum had AUC, sensitivity, and specificity values of 0.794, 69.2 and 87.3, respectively. In immunohistochemical study, expression of SOCS4 in EOC was lower than that in benign ovarian neoplasm. Serum exosomal miR-1290 could be considered as a biomarker for differential diagnosis of EOC from benign ovarian neoplasm and SOCS4 might be potential target gene of miR-1290 in EOC.

## 1. Introduction

Ovarian cancer is the second most common cancer among in women worldwide, accounting for about 2.5% of all cancers in women, but has the highest mortality rate of about 5 percent of all cancers; the 5-year survival rate of ovarian cancer is 90% in stage I and 75% in stage II, but less than 30% in stages III and IV [1]. Ovarian cancer, unlike other gynecologic cancers, is difficult to diagnose in early stages because it has few specific symptoms, and at the time of diagnosis more than 75% of the cases are found in advanced stages III or IV of the International Federation of Gynecology and Obstetrics (FIGO) stages [2]. As the death rate has not reduced by more than the incidence rate, this suggests that the improvements and advancements in ovarian cancer screening and treatment have only a modest impact in lowering ovarian cancer death rate.

A representative biomarker in the early screening of epithelial ovarian cancer is serum levels of cancer 125 (CA125). Since it was first described in 1983 by Bast et al. that CA125 was expressed increasingly in epithelial ovarian cancer, CA125 has been widely used as an early screening test for ovarian cancer [3]. However, it is elevated in more than 80% of patients with advanced stage ovarian cancer but only elevated in 50~60% of patients with stage I ovarian cancer, which is somewhat less sensitive in the early stage [4]. Although reproductive-age women with elevated levels of serum CA125 are more likely to develop malignant ovarian tumors, the usefulness of elevated levels of serum CA125 decrease in distinguishing malignant and benign conditions such as endometriosis, uterine fibroids, and pelvic inflammatory disease, pregnancy and menstruation [5]. To compensate for this, in 2009, Moore et al. described the risk of malignant ovarian cancer can be predicted by using ROMA (risk of malignancy) scores calculated from CA125 and human epididymis secretory protein 4(HE4) but Van Gorp et al. reported that the ROMA score is not superior for detecting ovarian cancer when compared to CA125 alone [6,7]. OVA1 is the first test cleared by the U.S. Food and Drug Administration (FDA) for aiding in the pre-surgical evaluation of a woman’s ovarian mass for cancer. According to a recent prospective clinical trial, OVA1 test was more sensitive in detecting ovarian cancer than clinical impression and CA125 [8]. Thus, there is a need for useful biomarkers to detect ovarian cancer in early stages. Exosomes are membrane-bounded extracellular vehicles, 30–100 nm in size, which are produced in endosomes of almost all eukaryotic cells [9,10]. Exosomes and extracellular vesicles are found in body fluids including blood (plasma or serum), urine, feces, ascites, etc. Cancer cells produce more exosomes than normal cells, and it is reported that cancer-derived exosomes can promote invasion and proliferation by intercellular communication in tumor microenvironment [10,11]. Recently, many biomolecules such as mRNAs, proteins, miRNAs have been identified in exosomes; therefore, researchers have had a great deal of interest in exosomes mainly because exosomes may play a role in cell to cell signaling through the transport of miRNAs, growth factors, and other small molecules [12]. miRNA-carrying exosomes secreted by tumor cells are likely to be non-invasive biomarkers and potential targetable factors [13]. Recent studies have shown that detection of specific exosomes could be a novel diagnostic tool. In fact, research is underway to apply exosome contents as biomarkers and capsules for therapeutic delivery [14,15]. The aim of the study was to identify serum exosomal miRNAs as a biomarker for early and differential diagnosis of EOC and we compared its expression both in tissues and exosome in serum of patients with EOC and benign ovarian neoplasm, and also tried to find out the expression of the target gene of the miRNA in tissues of EOC and benign ovarian neoplasm.

## 2. Materials and Methods

### 2.1. Serum and Tissues Specimens from Patients

All serum and tissue samples were obtained from patients who underwent primary cyto-reductive surgery or tumor resection for EOC and benign ovarian neoplasm, respectively, in Soonchunhyang University Cheonan Hospital and Gangnam Severance Hospital at Yonsei University between 2000 and 2019. Written informed consent was obtained from all patients. The present study was approved by the Institutional Review Board (IRB) of Soonchunhyang University Cheonan Hospital (IRB Number: 2019-10-013-008).

### 2.2. RNA Isolation and Assessment

Total RNA, including miRNA, was isolated from tissue samples using miRNeasy Mini kit (Qiagen, Hilden, Germany). Tissues were homogenized (IKA Works, Staufen, Germany) with 700 µL QIAzol lysis buffer (Qiagen). Homogenates were processed according to the manufacturer’s instructions. RNA was eluted with RNase-free water. The integrity of the RNA was confirmed using Agilent RNA 6000 Pico Kit and Small RNA Kit on Agilent 2100 Bioanalyzer (Agilent Technologies, Santa Clara, CA, USA).

### 2.3. Serum Exosomal RNA Isolation and Assessment

For exosomal RNA sequencing, serum samples from the patients with 3 benign ovarian neoplasm and 5 EOC were used. Exosomes were isolated from serum using ExoQuick isolation agent (System Bioscience, Palo Alto, CA, USA) in accordance with the manufacturer’s instructions. Serum samples (1000 μL) were centrifuged at 3000× *g* for 15 min to remove cells and cell debris. The supernatants were mixed with ExoQuick reagent (System Biosciences, Palo Alto, CA, USA) and incubated for 30 min at 4 °C. After incubation, the samples were centrifuged at 1500× *g* for 30 min to generate an exosome pellet that was resuspended in 100 μL of sterile phosphate-buffered saline (PBS). Total RNA was extracted from the exosomes using the miRNeasy Mini Kit (Qiagen, Hilden, Germany). Exosome suspensions were mixed with 700 μL QIAzol lysis buffer (Qiagen), and the mixtures were processed according to the manufacturer protocol. The RNA was eluted in 20 μL RNase-free water (Qiagen). The size distribution of purified RNA was assessed using an Agilent 2100 Bioanalyzer with an RNA Pico Chip and Small RNA Chip (Agilent Technologies, Santa Clara, CA, USA).

### 2.4. Small RNA Library Preparation and Sequencing

The samples were processed to produce exosomal RNA (10 ng) as the input for each library. Small RNA libraries were constructed using the SMARTer smRNA-Seq Kit for Illumina (Takara Bio, Kusatsu, Japan) following the manufacturer’s directions. Validation of libraries was performed using Agilent Technologies 2100 Bioanalyzer and DNA High Sensitivity Chips. We assessed the quantity of libraries using qPCR according to qPCR Quantification Protocol Guide (KAPA Library Quantification kits for Illumina Sequencing platforms). These libraries were qualified using TapeStation D1000 ScreenTape (Agilent Technologies, Waldbronn, Germany). Pooled libraries were sequenced on an Illumina HiSeq 2500 (Illumina, San Diego, CA, USA) for generating 101 bp single-end reads. Image decomposition and quality values calculation were performed using modules of the Illumina pipeline. Macrogen (Seoul, Korea) processed all steps for next-generation sequencing analysis.

### 2.5. Identification of Known and Novel miRNAs

Sequence alignment and detection of known and novel miRNAs were performed using miRDeep2 algorithm (Berlin Institute for Medical Systems Biology at the Max-Delbruck-Center for Molecular Medicine, Berlin-Buch, Germany). Prior to performing sequence alignment, *Homo sapiens* hg19 reference genome was retrieved from UCSC genome browser and indexed using Bowtie (1.2.3-07/05/2019; http://bowtie-bio.sourceforge.net/, accessed on 20 November 2021) to align sequencing reads to reference sequences. Sequence alignment was then performed for *Homo sapiens* matured and precursor miRNAs obtained from miRBase v21 (http://www.mirbase.org/, accessed on 20 November 2021).

### 2.6. Proportions of miRNAs and Other RNAs

Uniquely clustered reads were aligned to the reference genome, miRBase v21, and non-coding RNA database RNA central release 10.0 (https://rnacentral.org/, accessed on 20 November 2021) to classify known miRNAs and other types of RNAs, respectively.

### 2.7. Statistical Analysis of Differential miRNA Expression

Reads for miRNAs were subjected to Relative Log Expression (RLE) normalization with DESeq2 R library (Genome Biology Unit, European Molecular Biology Laboratory, Heidelberg, Germany). For preprocessing, mature miRNAs with zero counts across more than 60% of all samples were excluded. We added 1 with normalized read count of filtered miRNAs to facilitate log2 transformation to make the correlation plot. For each miRNA, logCPM (Counts Per Million) and log fold change were calculated between the test and control. A statistical hypothesis test for the comparison of the two groups was conducted using binomial Wald Test in DESeq2. |Fold change| ≥ 2 and *p* < 0.05 were used to identify differentially expressed miRNAs between two groups. Hierarchical clustering analysis using complete linkage and Euclidean distance was performed to display expression patterns of differentially expressed miRNAs that satisfied |fold change| ≥ 2 and *p* < 0.05. Differentially expressed genes were analyzed and visualized using R 3.6.1 (The R Foundation for Statistical Computing, Vienna, Austria).

### 2.8. miRNA Preparation and Validation by qRT-PCR

Two independent validations using qRT-PCR in FFPE and serum samples from the same patients were performed. In FFPE validation, 15 benign and 67 malignant FFPE samples were used. Total RNA, including miRNA, was extracted from Formalin-fixed, paraffin-embedded (FFPE) samples using miRNeasy FFPE kit (Qiagen, Hilden, Germany) according to the manufacturer’s instructions. In serum validation, 13 sera from benign patients and 71 sera from malignant patients were utilized for qRT-PCR. The RNA concentrations were quantified using a NanoDrop™2000 (Thermo, Waltham, MA, USA). Subsequently, RNA was reverse-transcribed using the TaqMan MicroRNA Reverse Transcription Kit (Applied Biosystems) for two upregulated (miR-1246 and miR-1290) miRNAs. The quantitative RT-PCR was performed on StepOnePlus^®^ Real-Time PCR System (Applied Biosystems) following the manufacturer’s recommendation with a standard relative quantification thermal cycling program. RNU48 (Applied Biosystems, Waltham, MA, USA) was served as an endogenous control. The relative expression level of each miRNA between two groups was determined by the −ΔΔCt method.

### 2.9. Cell Transfection & Inhibition of miR-1290

We used SKOV3-seeded cell density 5 × 10^5^ per well for 6-well cell culture plate and cultured in 37 °C CO_2_ incubator for 24 h miR-1290 inhibitor (mirVana miRNA inhibitor, Life Technologies, Carlsbad, CA, USA) and Lipofectamine RNAiMAX Transfection Reagent (Life Technologies, Carlsbad, CA, USA) were diluted in serum-free media of the 1:1 ratio. Prepared Transfection reagent-miR inhibitor solution was treated 250 μL per well. The cells were transfected by 25 pM of inhibitor in triplicate on 6-well cell culture plate and incubated for 72 h. Additionally, to exclude the influence of transfection reagents on gene expression, we used the untreated cell line SKOV3 as a control. Thereafter, transfected cells were treated with trypsin to harvest cell pellets.

### 2.10. miRNA Preparation and Validation by qRT-PCR

Total RNA was isolated from cell lines using the Hybrid-R™ RNA extraction kit (GeneAll, Seoul, Korea). Reverse transcription was performed using the ReverTra Ace^®^ qPCR RT kit (Toyobo, Osaka, Japan). Real time PCR was performed using the SYBR^®^ Green Real-time PCR Master Mix kit (Toyobo) and the following primer pairs: SOCS4 forward: 5′-ACC AAG AAA GGA AGC ACA GC-3′ and reverse 5′-TGA TCG AGG TGG GAA AGG AC-3′; and GAPDH forward: 5′-TGT TCG TCA TGG GTG TGA AC-3′ and reverse: 5′-GCA GGG ATG ATG TTC TGG AG-3′. The PCR cycle included one cycle at 95 °C for 3 min, followed by 40 cycles at 95 °C for 15 s, 60 °C at 15 s, and 72 °C for 25 s.

### 2.11. Western Blotting

Cells were washed in phosphate-buffered saline (PBS) and lysed in Pro-Prep™ protein extraction solution (INtRON, Seongnam, Korea). The lysate was centrifuged and the supernatant was denatured by boiling. The protein concentration was determined by the bicinchoninic acid (BCA) assay. Equal quantities of protein (30 µg/lane) were resolved by 10% sodium dodecyl sulfate-polyacrylamide gel electrophoresis (SDS-PAGE) and transferred onto an Immobilon polyvinylidene difluoride membrane (Millipore, Billerica, MA, USA). The membrane was then blocked for 1 h in 5% skim milk. Membranes were incubated overnight at 4 °C with anti-human SOCS4 antibody (Mybiosource, San Diego, CA, USA, MBS7043907) diluted 1:1000 and incubated with the diluted 1:1000 secondary antibody for 2 h at room temperature. The signal was detected using ECL Western detection reagents (Advansta, San Jose, CA, USA, K-12049-D50) and a Molecular Images were captured by CheBi (Cellgentek, Deajeon, Chemi-luminescence Bioimaging Instrument (Thermo Fischer, Waltham, MA, USA)).

### 2.12. Immunohistochemistry

Paraffin-embedded patient tissue blocks sectioned at 4 µm thickness. The slides were allowed to dry for a day and were left to warm at 60 °C degrees for an hour. For antigen retrieval, 3% H_2_O_2_ and 95 °C antigen retriever buffer were used. For permeability, 0.2% triton solution was treated and for blocking, 5% BSA in PBS was treated for 15 min. The 1st antibody was treated with Rabbit Polyclonal antibody SOCS4 (1:100, Mybiosource, MBS7043907) and incubated at 4 °C overnight. Additionally, Goat anti-Rabbit IgG (H + L), HRP (1:100, Thermofisher, A11008) was treated as a secondary antibody for 1 h at room temperature. Stained with DAB Substrate Kit (3,3′-diaminobenzidine, VECTORLABS, SK-4100) and counterstaining with 50% Hematoxylin for 30 s. Then, the slide was dried at 37 °C for 1 h (Mounted with Eukitt^®^ Quick-hardening mounting medium (Sigma Aldrich, St. Louis, MO, USA, 03989-100)). Slides were interpreted twice by two pathologists.

### 2.13. Statistical Analysis

Statistical evaluation was performed with IBM^®^ SPSS Statistics 21 (Chicago, IL, USA) and GraphPad Prism Software version 6.0 (San Diego, CA, USA). Statistical relevance of the relative expression between benign and malignant samples was analyzed by the unpaired *t* test. The values were presented as the median, the interquartile range and the standard deviation. A *p*-value ≤ 0.05 was considered to be statistically significant. Receiver operating characteristic (ROC) curve analysis and the area under the curve (AUC) were used to assess diagnostic performance for each biomarker individually (estimate the feasibility of using the serum exosomal miRNA expression levels as diagnostic markers for discriminating OC patients from benign patients). The results were presented as odds ratios with 95% confidence intervals and *p*-values.

## 3. Results

### 3.1. Baseline Clinical Characteristics

First, we have confirmed the baseline clinical characteristics of the patients with EOC group and benign ovarian neoplasm group. Most of the histology in malignancy group and benign ovarian neoplasm group is high-grade serous carcinoma and serous cystadenoma, respectively. The median age was 51 years (range 24–81). 33 and 30 (44.26 and 42.25%) patients were FIGO stage I and II, 34 and 41 (50.75 and 57.75%) patients were FIGO stage ≥ III at diagnosis from FFPE and serum samples (Table 1 and Table 2). Tumor marker CA125 was above normal range around 80% in EOC group and over 25% in benign ovarian neoplasm group (*p* < 0.001).

### 3.2. Differentially Expressed miRNAs in Malignant Ovarian Cancer Patients Based on RNA Sequencing: Identification of Candidate miRNAs

**Figure 1:**
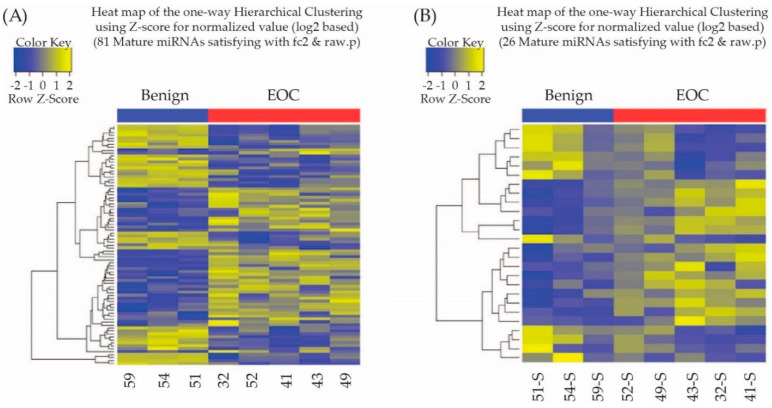
(**A**) Heatmap showing z score of miRNAs from benign tumors (*n* = 3) and malignant tumors (*n* = 5) with 44 upregulated (yellow) and 37 downregulated (blue) miRNAs. (**B**) Heat map showing z score of exosomal miRNAs from serum of benign tumors (*n* = 3) and malignant tumors (*n* = 5) with 15 upregulated (yellow) and 11 downregulated (blue) miRNAs.

To investigate the tissue origin of miRNAs, we compared the miRNA expression between matched serum and tissue samples. Based on this, we identified 81 miRNAs (44 upregulated and 37 downregulated miRNAs) in the tissues of six patients with EOC compared with patients with benign ovarian neoplasm. Furthermore, a total of 26 exosomal miRNAs (15 upregulated and 11 downregulated miRNAs) were identified as differentially expressed between sera of EOC group and benign ovarian neoplasm group (|Fold change| ≥ 2 and *p* < 0.05; Figure 1).

**Figure 2:**
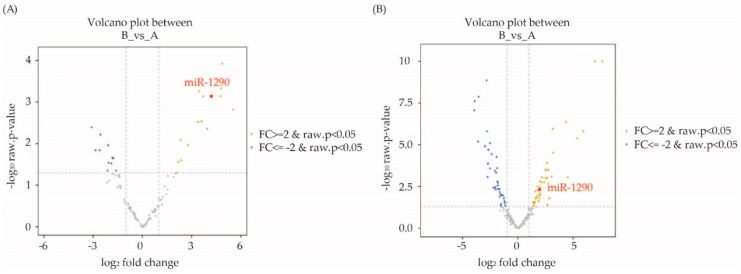
(**A**) Serum. (**B**) FFPE: Volcano plot showing differences in microRNAs (miRNAs) expression between benign and malignant ovarian cancer patients. Yellow dots represent upregulated miRNAs and blue dots represent downregulated miRNAs.

Results are also shown in a volcano plot from serum and tissue samples (Figure 2). Only seven miRNAs (hsa-miR-1246, hsa-miR-1290, has miR-21-5p, hsa-miR-7-5p, hsa-miR-93-5p, hsa-miR-16-5p and hsa-miR-29c-3p) were identified as differentially expressed in both matched tissue and serum samples (Table 3).

### 3.3. Validation of Candidate miRNAs by qRT-PCR

**Figure 3:**
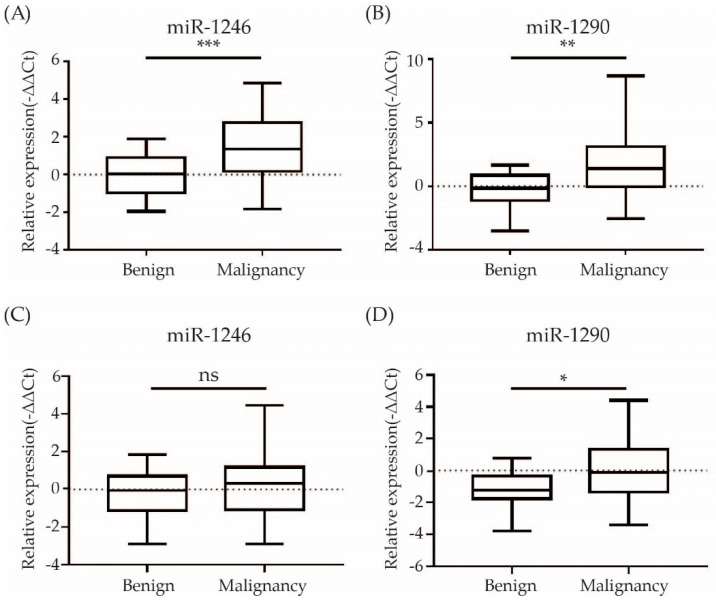
Evaluation of the two selected miRNA expression values in FFPE and serum samples by quantitative RT-PCR (**A**,**B**) FFPE: A total of 45 (15 benign ovarian neoplasm, 67 EOC) (**C**,**D**) Serum: A total of 84 (13 benign ovarian neoplasm, 71 EOC) were used for qRT-PCR. * *p* < 0.05, ** *p* < 0.01, *** *p* < 0.001. ns: not significant.

From the differentially expressed miRNAs, we chose to focus on miR-1246 and miR-1290 which were the topmost upregulated miRNAs among common miRNAs between tissue and serum exosome. To demonstrate the expression levels of candidate miRNAs to ovarian cancer tissues and sera, their levels were examined by qRT-PCR in FFPE and sera of patients with EOC. Both miR-1246 and miR-1290 showed consistent upregulation in EOC FFPE samples (*n* = 67) compared with benign ovarian neoplasm FFPE samples (*n* = 15) (*p* < 0.01 for miR-1246 and miR-1290). In serum, the expression level of miR1246 and miR-1290 was higher in EOC patients (*n* = 71) compared to benign ovarian neoplasm patients (*n* = 13). The *p*-value of miR-1290 was 0.0005 but miR-1246 was not significant (Figure 3).

### 3.4. Diagnostic Value of Exosomal miRNA 1290 for Epithelial Ovarian Cancer

**Figure 4:**
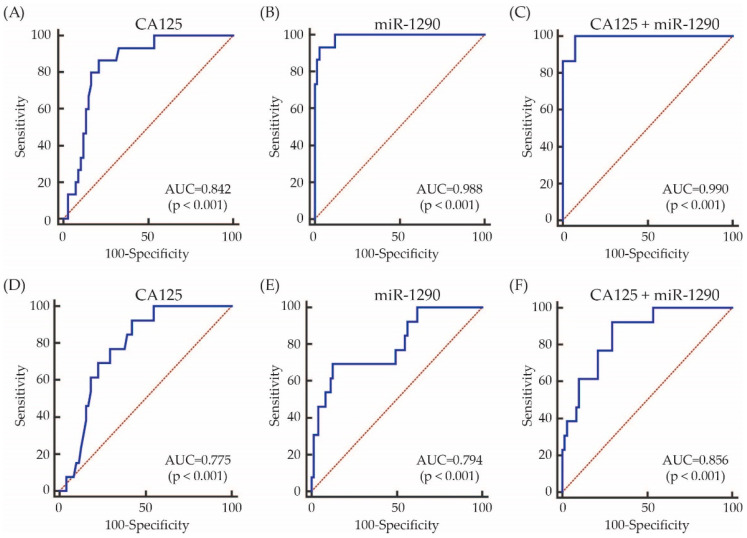
ROC curves for the identification of patients with EOC vs. benign ovarian neoplasm controls based on the expression of CA125, miR-1290, and the combination of both. The AUC values are shown on the graphs. (**A–C**) FFPE, (**D**–**F**) Serum.

The diagnostic performance value of serum miR-1290 was calculated by ROC curve analysis to discriminate EOC group from benign ovarian neoplasm group. Using a cut-off of 1.71, the miR-1290 from tissue had AUC, sensitivity, specificity positive predictive (PP) and negative predictive (NP) values of 0.988, 93.3, 97.0, 87.5 (0.616–0.985) and 98.5% (0.918–1.000), respectively. Using a cut-off of 0.73, the exosomal miR-1290 from serum had AUC, sensitivity, specificity, PP and NP values of 0.794, 69.2, 87.3, 50 (0.26–0.74) and 94% (0.852–0.983), respectively (Figure 4 and Supplementary Figure S3). Although the diagnostic performance of serum exosomal miRNA-1290 was not better than CA125, the combination of serum exosomal miR-1290 and CA125 significantly improved AUC value from 0.775 to 0.856 compared to serum exosomal miR-1290 alone (*p* < 0.001; Figure 4); however comparing to CA125 alone, neither serum exosomal miR-1290 alone nor the combination of serum exosomal miR-1290 and CA125 improved AUC value (Supplementary Figure S1).

### 3.5. Decreased Expression of SOCS4 in Malignancy Group That Was Negatively Regulated by miRNA 1290

**Figure 5:**
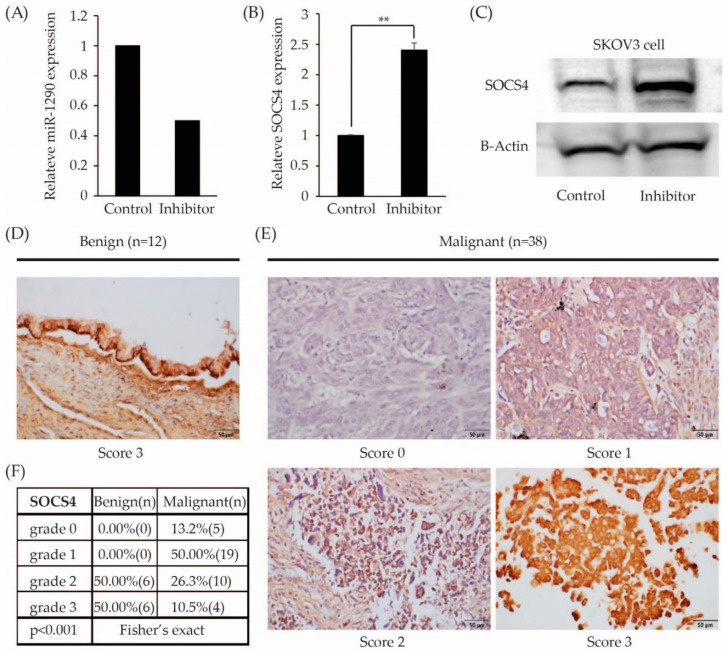
SOCS4 was a target of miR-1290. (**A**) The miR-1290 expression level was decreased after treatment of miR-1290 inhibitor in SKOV3 cell lines by RT-qPCR. (**B**,**C**) SOCS4 expression was increased in miR-1290 inhibitor treated SKOV3 cells by RT-qPCR and Western blot. B-Actin was used as the loading control. Representative images of nuclear SOCS4 immunohistochemistry staining in EOC and benign ovarian neoplasm (scale bar = 50 μm). (**D**) Benign ovarian neoplasm and (**E**) Ovarian cancer, detection of SOCS4 protein expressions in patients’ tissues via immunohistochemistry method. (**F**) The expression level of SOCS4 in ovarian cancer tissues is significantly decreased compared with that of benign ovarian tumor. SOCS, suppressor of cytokine signaling. ** *p* < 0.0001.

To determine the targets of miR-1290 were used web tools include Targetscan and miRanda, SOCS4 was selected as a potential target of miR-1290. After treatment with miR-1290 inhibitor, miR-1290 expression level in SKOV3 cells was decreased (Figure 5A) whereas mRNA and protein expression of SOCS4 was significantly increased in SKOV cells (*p* < 0.001; Figure 5B,C and Supplementary Figure S2). In the present study, immunohistochemistry revealed that expression of SOCS4 decreased significantly in EOC group (*n* = 38) compared to benign ovarian neoplasm group (*n* = 12) (*p* < 0.001; Figure 5D,F). In benign ovarian neoplasm group, there were no patients with grade 0 or grade 1 SOCS4 expression, while 63% of EOC has grade 0 or grade 1 expression.

## 4. Discussion

In this study, based on RNA sequencing, analysis of serum exosomal miRNA expression profiles revealed that three miRNAs including hsa-miR-1246, hsa-miR-1290 and hsa-miR-29c-3p exhibited the same regulation direction in both tumor tissues and sera. We think it is extremely important that if certain exosomal miRNA increases in the serum, same miRNA should increase in the cancer tissue. Therefore, we performed qRT-PCR to detect expression levels of hsa-miR-1246, hsa-miR-1290 and hsa-miR-29c-3p both in the serum and the tissue of patients with epithelial ovarian cancer (EOC) and benign ovarian neoplasm, and we confirmed that the expression of tissue miR1246 and miR-1290 were statistically higher in patients with EOC than in patients with benign ovarian neoplasm, whereas the expression of serum exosomal miR-1290 was statistically higher in patients with EOC than in patients with benign ovarian neoplasm. Serum and tissue miR-1290 was significantly elevated in patients with EOC compared to patients with benign ovarian neoplasm and could discriminate malignancy versus benign ovarian neoplasm with an area under the curve (AUC) of 0.932 and 0.812, respectively, suggesting its potential as both a useful biomarker for differential diagnosis and prognostic factor. The combination of miR-1290 and CA125 significantly improved the AUC value from 0.812 to 0.95.4. The OVA1 test seems to be more sensitive than CA125 or miR-1290 in the preoperative clinical setting; however, a higher false positive rate is still an unresolved issue.

In 2008, Taylor et al. reported eight exosomal miRNAs (miR-21, miR-141, miR-200a, miR-200b, miR-200c, miR-203, miR-205, miR-214) extracted from serum of ovarian cancer patients. It was reported for the first time that there was an increase compared to benign ovarian tumors. In that study, circulating tumor exosomes were isolated using an anti-EpCAM-modified MACS procedure, and the microRNA profile of ovarian tumors was compared with that of tumor exosomes isolated from the same patient. These results mean that circulating miRNA profiles accurately reflects the tumor profiles [16]. In a cohort of 56 high-grade serous ovarian cancer (HGSOC) patients, Shah et al. reported that Serum miR-375, along with Ca125, is a biomarker that discriminates between normal women and patients with ovarian cancer [17].

Kobayashi et al. also reported that miR-1290 is a specific discriminator for HGSOC suggesting the potential of this miRNA as a biomarker for HGSOC. However, they did not use next generation RNA sequencing but used miRNA microarray to detect candidate miRNAs, and they validated these miRNAs not in tumor tissues from patients but only in cell lines [18].

miR-1290 was first reported in human embryonic stem cells [19]. It was reported that upregulation of miR-1290 was associated with progression of various cancers, including pancreatic cancer [20], esophageal squamous cell carcinoma [21] and colon cancer [22]. Zhang et al. indicated that miR-1290 was a tumor-initiating, cell-specific RNA, which were crucial drivers of tumor initiation and progression in non-small cell lung cancer (NSCLC) [23]. A previous study reported that miR-1290 was upregulated in tissues and serum samples from patients with lung adenocarcinoma and correlated with poor prognosis, and suppressor of cytokine signaling 4 (SOCS4) was target of miR-1290, by targeting SOCS4, miR-1290 facilitated the JAK/STAT3 and PI3K/AKT pathways [24].

SOCS family is a group of cytokine-inducible negative regulators by inhibiting multiple signaling pathways, especially the JAK/STAT signaling pathway [25,26]. SOCS4 was previously shown to be associated with earlier tumor stage and better clinical prognosis in breast cancer [27].

In this study, the expression levels of SOCS4 were examined in EOC and benign ovarian neoplasm. Decreased expression of SOCS4 was shown in EOC compared to benign ovarian neoplasm. Although we did not undertake clinic pathological analysis due to limited patients’ medical information and small sample size, to our knowledge this is first report that showed difference of SOCS4 expression in EOC and benign ovarian neoplasm. Several limitations should be acknowledged. The sample size is too small to reach a solid conclusion. Large and prospective registry-embedded trials would be needed to strengthen our hypothesis that serum miR-1290 can serve as a biomarker of EOC. Additionally, the mechanisms and potential pathways between miR-1290 and other target genes, including SOCS4 in EOC, need to be explored. Despite the small sample size, our results show that miR-1290 might be a useful biomarker which can discriminate epithelial ovarian cancer from benign ovarian neoplasm. Further studies would be required to explore other target genes of miR1290 and to identify the true target genes of miR-1290 in EOC, in order to clarify its role.

## Figures and Tables

**Table 1:** Characteristics of the FFPE sample from patients.

FFPE	Total (*n* = 82)	Type		*p*-Value
		Benign (*n* = 15)	EOC (*n* = 67)	
**Age (years, mean ± SD)**	51.80 ± 11.74	46.27 ± 15.72	53.04 ± 10.4	0.1297
**FIGO stage (%)**	***n*** **(*%*)**	***n*** **(*%*)**	***n*** **(*%*)**	
Stage1	26 (38.81)	-	26 (38.81)	-
Stage2	7 (10.44)	-	7 (10.44)	
Stage3	26 (38.81)	-	26 (38.81)	
Stage4	8 (11.94)	-	8 (11.94)	
**CA 125 (U/mL)**				
≥35	60 (73.17)	4 (26.67)	56 (83.58)	<0.001
<35	22 (26.83)	11 (73.33)	11 (16.42)	
**miR-1290**				
≥1.71	66 (80.49)	65 (97.01)	1 (6.67)	<0.001
<1.71	16 (19.51)	2 (2.99)	14 (93.33)	

**Table 2:** Characteristics of the serum sample from patients.

Serum	Total (*n* = 84)	Type		*p*-Value
		Benign (*n* = 13)	EOC (*n* = 71)	
**Age (years, mean ± SD)**	51.81 ± 11.66	45.46 ± 14.36	52.97 ± 10.82	0.0933
**FIGO stage (%)**	***n*** **(*%*)**	***n*** **(*%*)**	***n*** **(*%*)**	
Stage1	25 (35.21)	-	25 (35.21)	>0.99
Stage2	5 (7.04)	-	5 (7.04)	
Stage3	34 (47.89)	-	34 (47.89)	
Stage4	7 (9.86)	-	7 (9.86)	
**CA 125 (U/mL)**				
≥35	64 (76.19)	6 (46.15)	58 (81.69)	0.011
<35	20 (23.81)	7 (53.85)	13 (18.31)	
**miR-1290**				
≥0.73	66 (78.57)	4 (30.77)	62 (87.32)	<0.001
<0.73	18 (21.43)	9 (69.23)	9 (12.68)	

**Table 3:** Fold change of miRNAs expressions in EOC group compared to benign ovarian neoplasm group.

Mature miRNA	Fold Change in Tissue	Fold Change in Serum
has-miR-1246	6.78	27.61
has-miR-1290	3.66	27.04
has-miR-21-5p	4.1	−3.43
has-miR-7-5p	41.33	−4.27
has-miR-93-5p	3.76	−4.39
has-miR-16-5p	2.63	−8.58
has-miR-29c-3p	−2.4	−3.05

## Data Availability

Not applicable.

## Supplementary Materials

The following supporting information can be downloaded at: https://www.mdpi.com/article/10.3390/cimb44010021/s1, Figure S1: The comparison diagnostic performance of miR-1290 with CA125 alone (A), CA125 with the combination (B), and miR-1290 with the combination (C). Figure S2: SOCS4 Western blotting full image. Figure S3: The dot plot data for the identification of patients with EOC vs. benign ovarian neoplasm controls based on the expression of CA125 and miR-1290. (A,B) FFPE, (C,D) Serum.
