# Supplementary material for: Circulating Exosomal miR-1290 for Diagnosis of Epithelial Ovarian Cancer
Source: Curr Issues Mol Biol. 2022 Jan 9;44(1):288–300. doi: 10.3390/cimb44010021 (PMC8928998; doi:10.3390/cimb44010021)
Supplement: Supplementary file 1 [file cimb-44-00021-s001.zip › cimb-1500812-supplementary.pdf]

current issues in
molecular biology

MDPI

Article

Circulating Exosomal miR-1290 for Diagnosis of Epithelial Ovarian Cancer

Hyeji Jeon [1,2,†], SuMin Seo [3,*,†], Tae Wan Kim [2,4], Jaesung Ryu [2,4], Hyejeong Kong [2,4], Si Hyeong Jang [5], Yong Soo Jang [1], Kwang Seock Kim [2], Jae Hoon Kim [6], Seongho Ryu [3,*] and Seob Jeon [1,2,*]

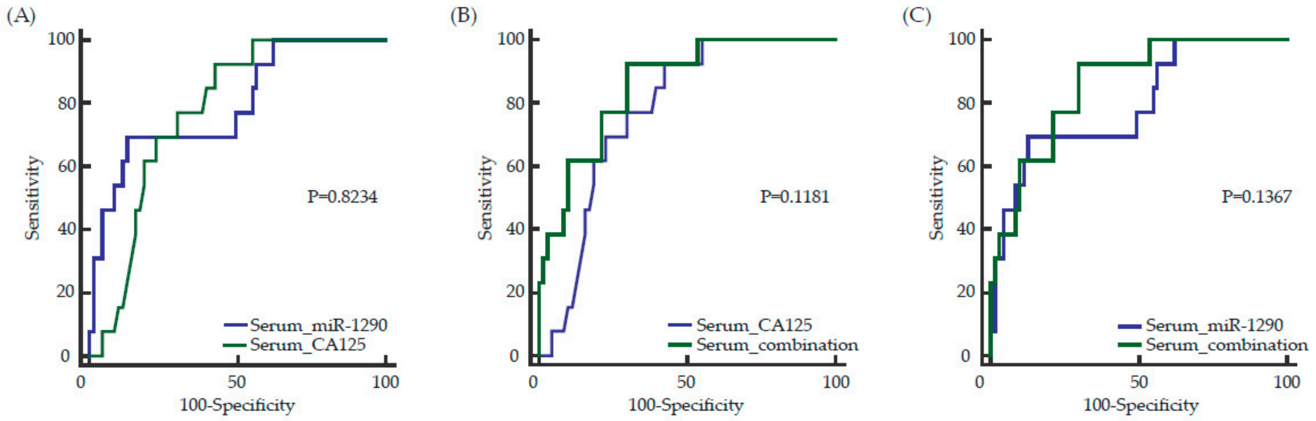

Figure S1. The comparison diagnostic performance of miR-1290 with CA125 alone (**A**), CA125 with the combination **(B)**, and miR-1290 with the combination (**C**).

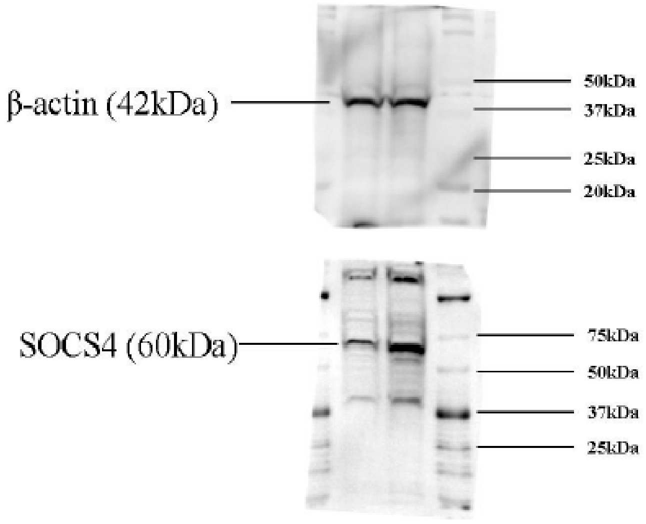

Figure S2. SOCS4 Western blotting full image.

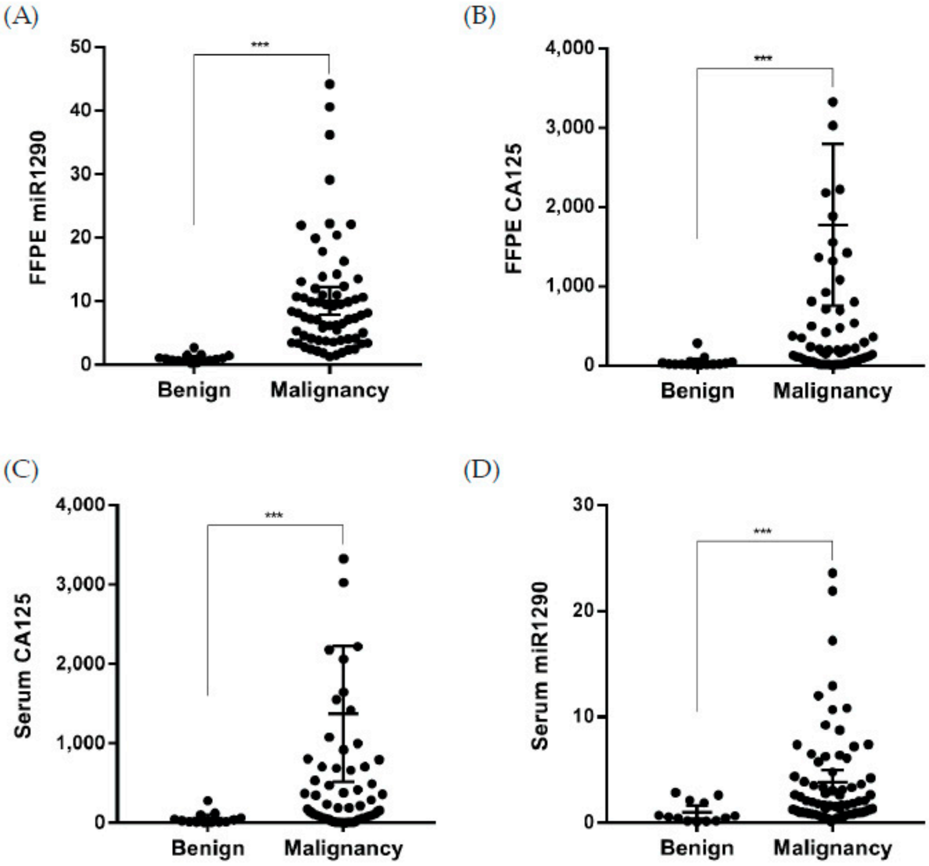

Figure S3. The dot plot data for the identification of patients with EOC vs. benign ovarian neoplasm controls based on the expression of CA125 and miR-1290. (**A**,**B**) FFPE, (**C**,**D**) Serum.
